# Supplementary material for: Ammonium Polyphosphate with High Specific Surface Area by Assembling Zeolite Imidazole Framework in EVA Resin: Significant Mechanical Properties, Migration Resistance, and Flame Retardancy
Source: Polymers (Basel). 2020 Mar 2;12(3):534. doi: 10.3390/polym12030534 (PMC7182838; doi:10.3390/polym12030534)
Supplement: Supplementary file 1 [file polymers-12-00534-s001.pdf]

Supplementary Materials

Ammonium Polyphosphate with High Specific Surface Area by Assembling Zeolite Imidazole Framework in EVA Resin: Significant Mechanical Properties, Migration Resistance and Flame Retardancy

Jingyu Wang, Hui Shi, Pinlie Zhu, Yuanjie Wei, Jianwei Hao*

[1] School of Materials Science and Engineering, Beijing Institute of Technology, Beijing 100081, China; quillwang@163.com (J.W.); shihuia@mail.ustc.edu.cn (H.S.); 15636125761@163.com (P.Z.); weiyuanjia21@163.com (Y.W.); hjw@bit.edu.cn (J.H.)

[2] National Engineering Technology Research Center of Flame Retardant Materials, Beijing 100048, PR China

* Corresponding author: hjw@bit.edu.cn ; Telephone Numbers: 0086 10 68913075

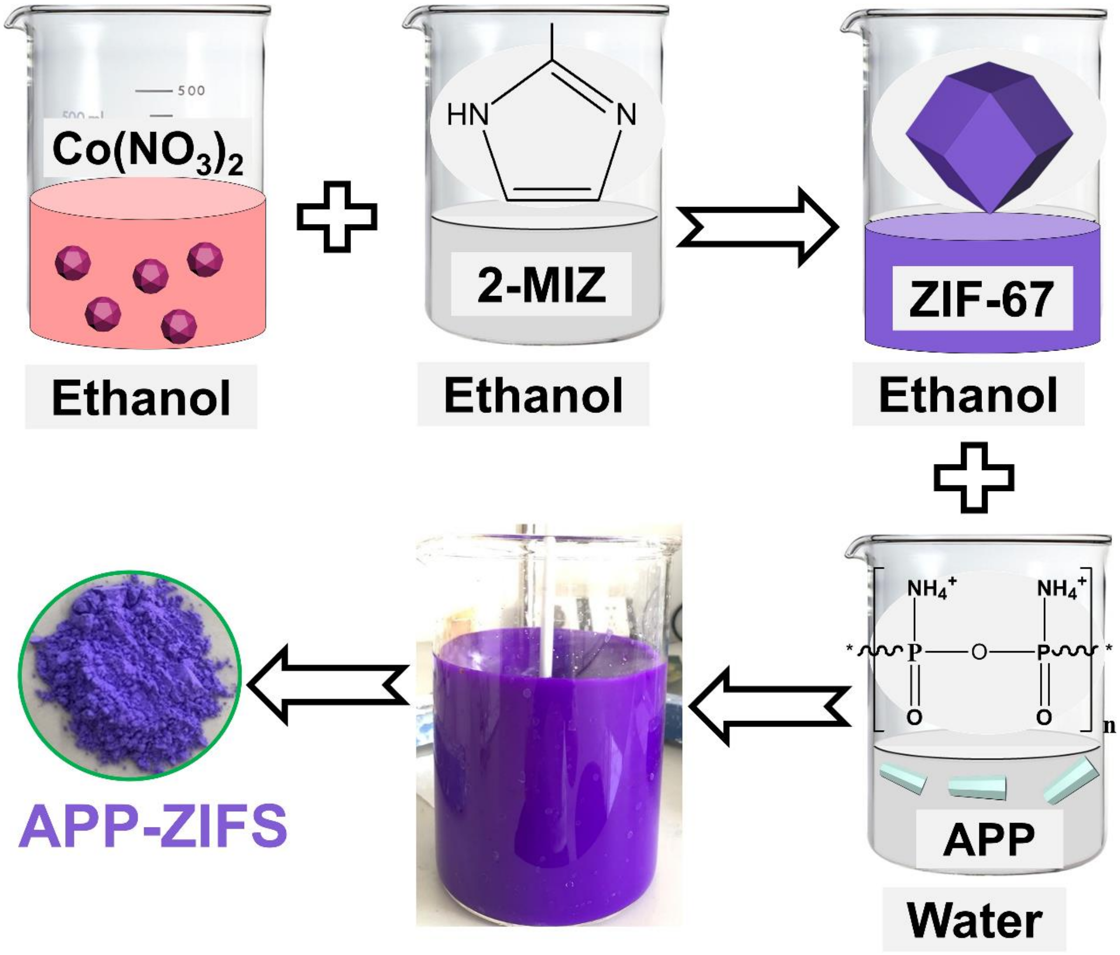

Figure S1. Illustration of the interface assembly procedure of APP-ZIFs

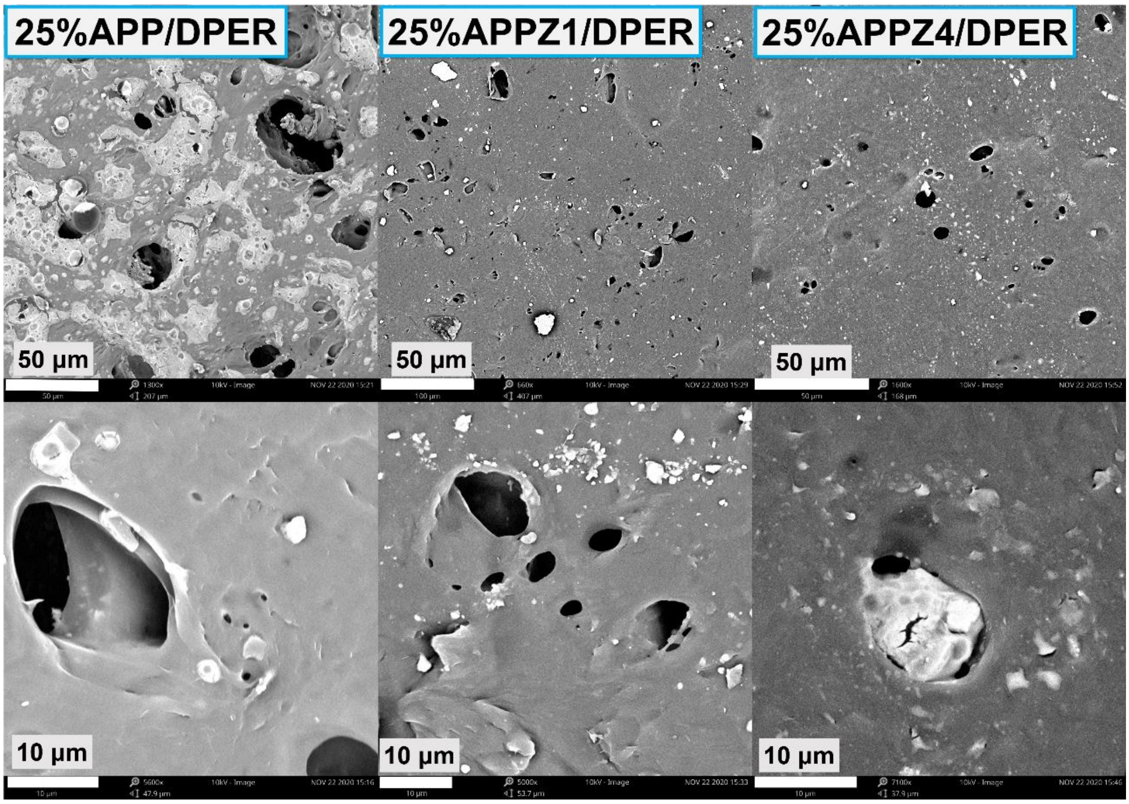

Figure. S2. Surface micromorphology of EVA composites after hot water treatment tests (9 days)

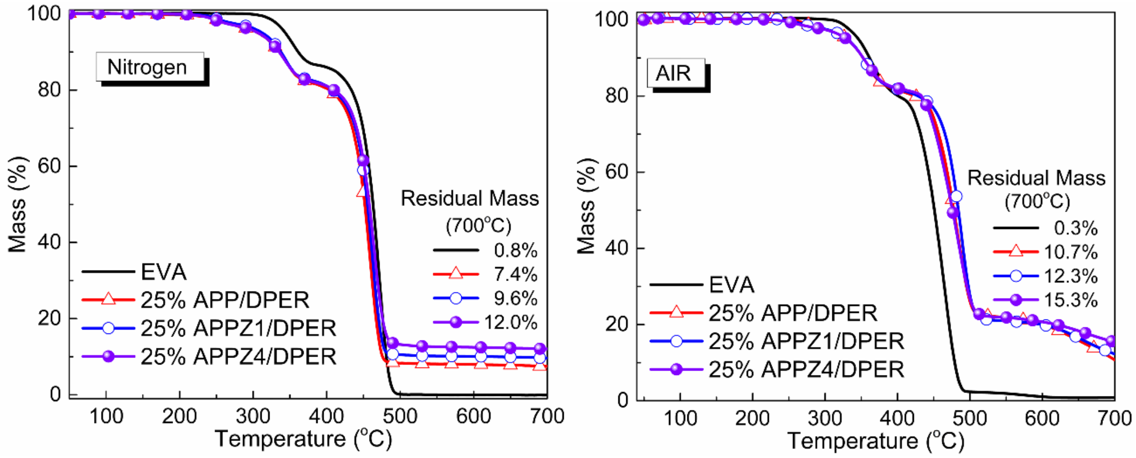

Figure. S3. TG curves of EVA composites in nitrogen and air atmosphere

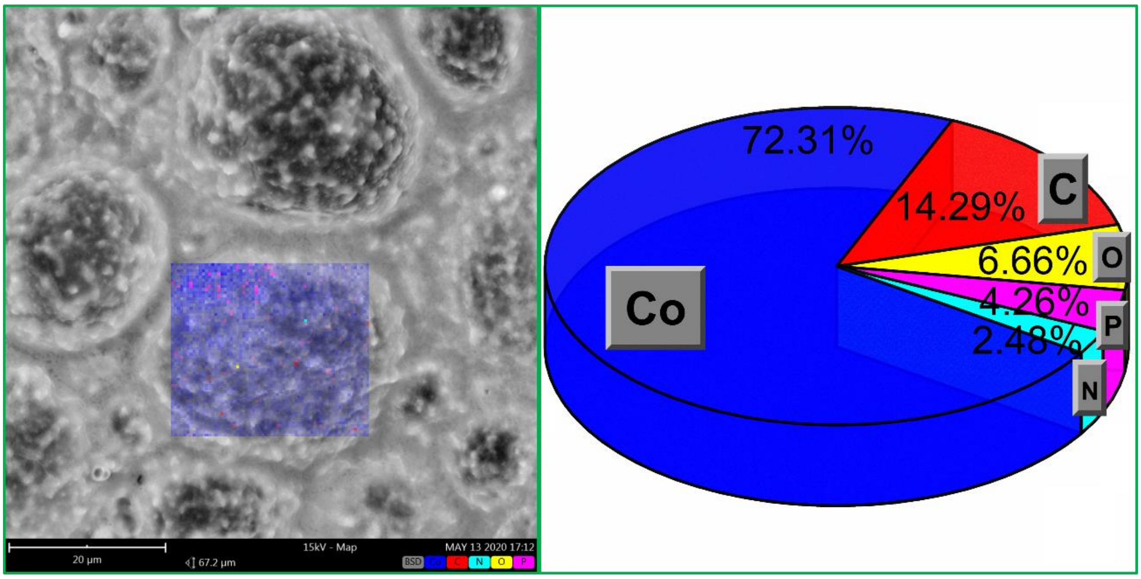

Figure S4. Elemental composition of the assemble frameworks in 25% APPZ4/DPER surface char by EDS

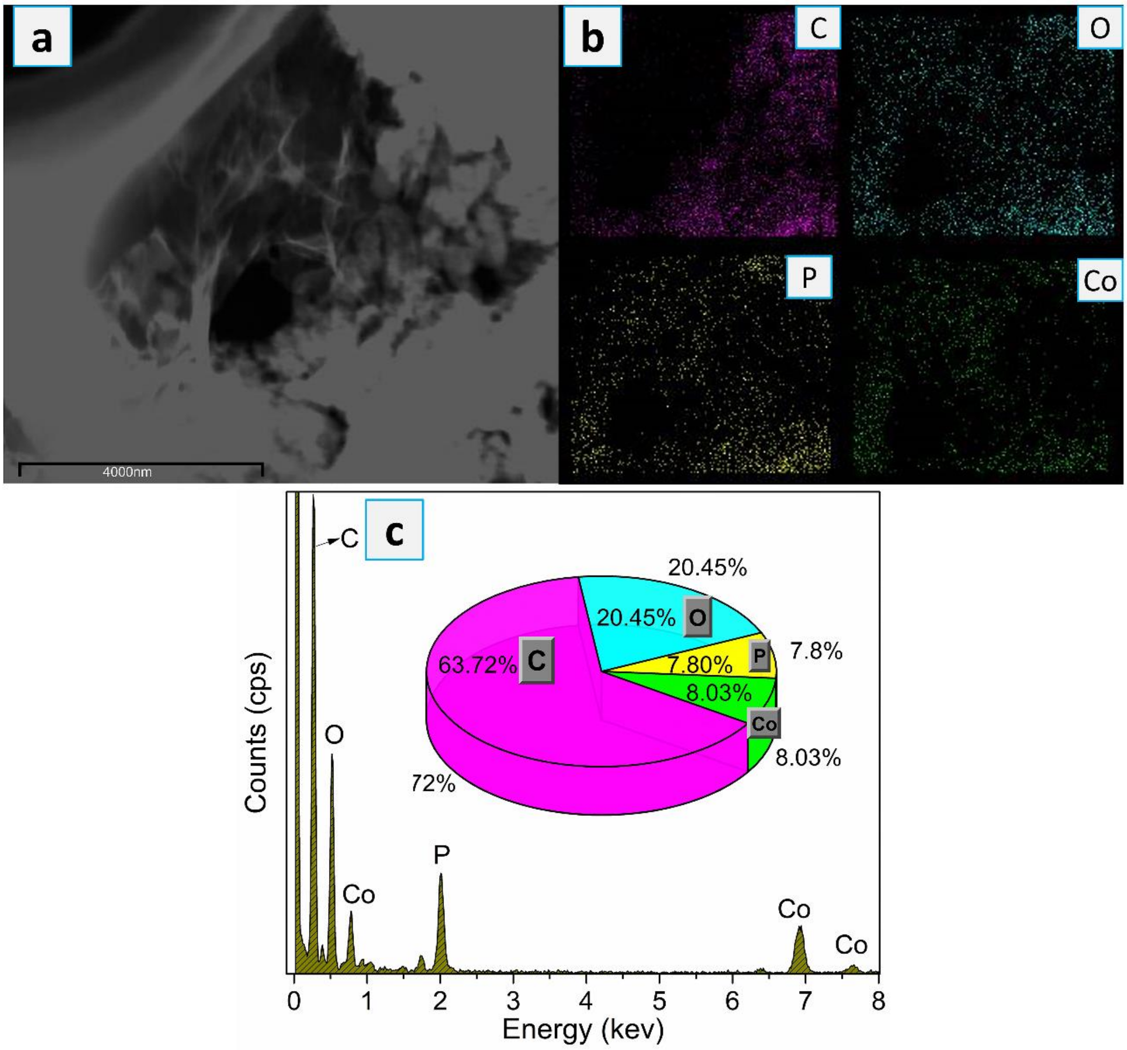

Figure S5. TEM photo of 2D nano film structure (a), EDS mapping photos of the C, O, P and Co elements; elemental composition by EDS (c).

Table S1 Formulations of EVA Composites and Control EVA

Samples	EVA18 (wt. %)	gEVA (wt. %)	APP (wt. %)	APPZ1 (wt. %)	APPZ4 (wt. %)	DPER (wt. %)
EVA	95.00	5.00	--	--	--	--
25%APP/DPER	70.00	5.00	18.75	--	--	6.25
28%APP/DPER	67.00	5.00	21.00	--	--	7.00
30%APP/DPER	65.00	5.00	22.50	--	--	7.50
25% APPZ1/DPER	70.00	5.00	--	18.75	--	6.25
25% APPZ4/DPER	70.00	5.00	--	--	18.75	6.25
